# Tuberous sclerosis complex associated lymphangioleiomyomatosis

**DOI:** 10.1093/qjmed/hcad125

**Published:** 2023-06-07

**Authors:** Z Han, X Xue, J Wang, D Lu

**Affiliations:** Department of Gerontology, The First Affiliated Hospital of Shandong First Medical University and Shandong Provincial Qianfoshan Hospital, Jinan, China; Department of Gerontology, The First Affiliated Hospital of Shandong First Medical University and Shandong Provincial Qianfoshan Hospital, Jinan, China; Department of Gerontology, The First Affiliated Hospital of Shandong First Medical University and Shandong Provincial Qianfoshan Hospital, Jinan, China; Department of Respiratory, The First Affiliated Hospital of Shandong First Medical University and Shandong Provincial Qianfoshan Hospital, Shandong Institute of Respiratory Diseases, Shandong Institute of Anesthesia and Respiratory Critical Medicine, Jinan, China

A 32-year-old Chinese woman was admitted with a chief complaint of intermittent haemoptysis and dyspnoea for 2 years. She had suffered from epilepsy for many years and right nephrectomy was performed 6 years ago to remove a giant mass, pathology showed angiomyolipoma. Physical examination revealed focal hypopigmentation, shark-skin-like spots, facial angiofibromas, fibrous plaques and ungual fibromas. Brain computed tomography (CT) revealed multiple calcified subependymal nodules. Chest CT revealed multiple air-filled cysts and nodules of variable sizes bilaterally (Figure 1a). Abdominal CT showed multiple hamartomas of the liver and left kidney (Figure 1b). Pulmonary function tests (PFTs) showed a normal ventilation function, and a decreased diffusing capacity of carbon monoxide of 5.676 mmol/min/kPa (65.77% predicted). Next-generation and Sanger sequencing identified a novel TSC2 frameshift variant (NM_000548.5: exon29: c.3322del: p. A1108Rfs*83) in this case. Based on these findings, the diagnosis of tuberous sclerosis complex associated lymphangioleiomyomatosis (TSC-LAM) was established. She was treated with the mechanistic target of rapamycin (mTOR) complex 1 inhibitor sirolimus at 1 mg per day, and her symptoms partially improved.

**Figure 1.**
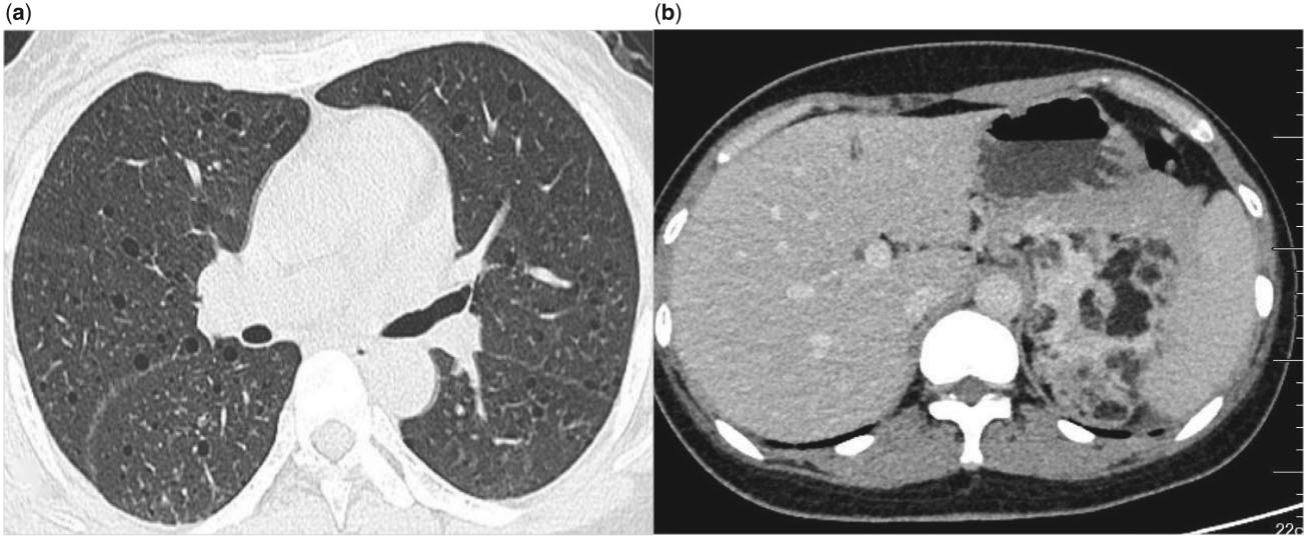
(**a**) Chest CT scans showing multiple thin-walled, round, well-defined cysts and nodules of variable sizes bilaterally. (**b**) Abdominal CT showing multiple hamartomas of the liver and left kidney.

Lymphangioleiomyomatosis (LAM) is a rare, chronic progressive, low-grade malignant tumours that affect women almost exclusively and characterized by causing cystic destruction of the lung parenchyma due to infiltration of LAM cells. Sporadic LAM (S-LAM) and TSC-LAM are the two forms of LAM identified clinically, the natural history of the two LAM subgroups is similar,^1^ and both of which are caused by a single inactivating mutation in the TSC gene. Mutations in TSC1 or TSC2 genes cause hamartolin or nodulin protein loss of function, resulting in overactivation of the mTOR pathway, and then leading to the recruitment of LAM cells, clonal origins for neoplastic cells, dysregulated proliferation, enhanced invasion and migration and lymphangiogenesis.^2^ The most frequent clinical features of LAM are exertional dyspnoea and recurrent pneumothoraces. Airflow obstruction and decreased lung diffusion capacity are the most commonly observed abnormalities of PFTs in LAM patients.^3^ On high-resolution computed tomography (HRCT), LAM is characterized by diffusely scattered thin-walled, round, well-defined cysts or multifocal micronodular pneumocyte hyperplasia, which is another manifestation of TSC-LAM not observed in S-LAM patients.^4^ Vascular endothelial growth factor D (VEGF-D) is a useful diagnostic and prognostic biomarker, which reflects the severity of the disease and decreases after sirolimus treatment.^5^ Sirolimus, an mTOR inhibitor, is the first-line treatment for patients with LAM, its effects have been well established and was widely recommended by international guideline.^6^ LAM complicating pneumothorax or chylothorax should be treated surgically, and end-stage LAM requires lung transplantation. In conclusion, raising awareness of the disease and increasing early intervention can delay disease progression.

## Ethics approval and consent to participate

All participants signed written informed consent forms and the study was approved by the Ethics Committee of the First Affiliated Hospital of Shandong First Medical University and Shandong Provincial Qianfoshan Hospital (XMSBLL2022(400)).

## Consent for publication

All related medical data and genetic testing results are released with the written consent of all participants or the legal guardians of participants under the age of 18. A copy of the written consent form can be obtained from the journal publisher. All authors gave their consent for publication of this manuscript.

## Availability of data and materials

All data generated or analyzed during this study would be available from the corresponding authors on reasonable request.

## Supplementary Material

hcad125_Supplementary_Data
