# Supplementary figures and images for: Tuberous sclerosis complex associated lymphangioleiomyomatosis
Source: QJM. 2023 Jun 7;116(10):873–4. doi: 10.1093/qjmed/hcad125 (PMC10593382; doi:10.1093/qjmed/hcad125)

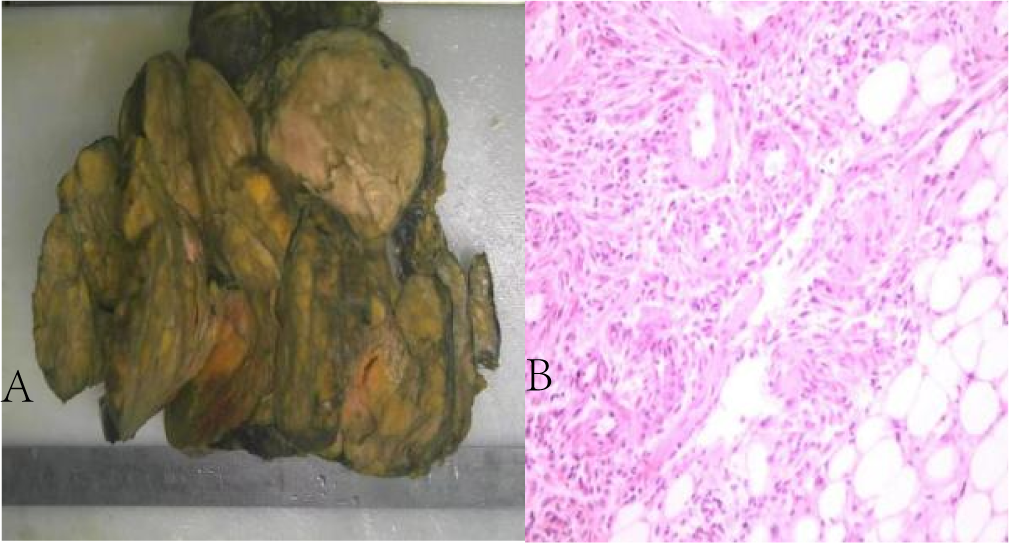

Supplement: hcad125_Supplementary_Data [file hcad125_supplementary_data.zip › suppl_data/sFigure1.tif]

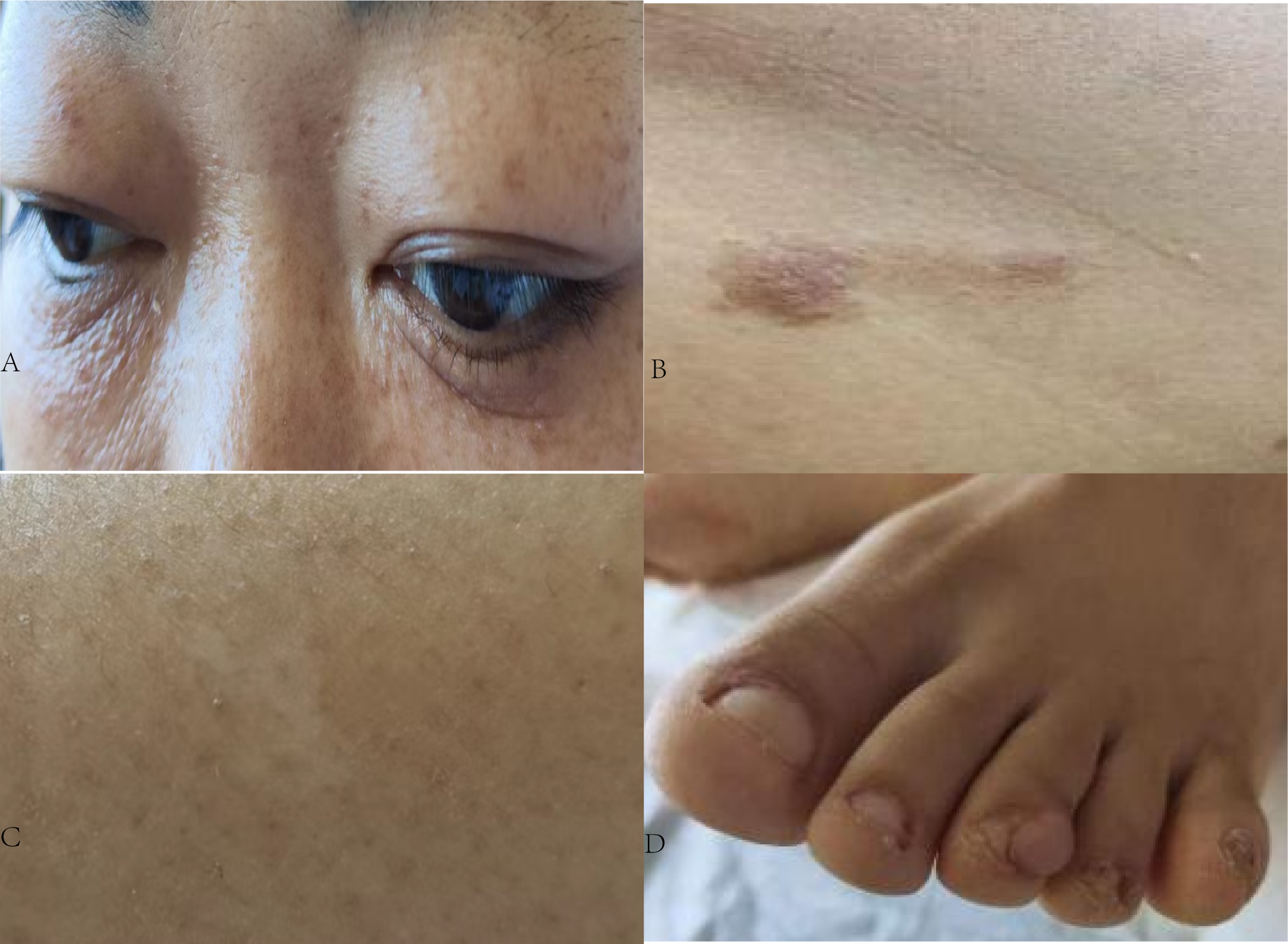

Supplement: hcad125_Supplementary_Data [file hcad125_supplementary_data.zip › suppl_data/sFigure2.tif]

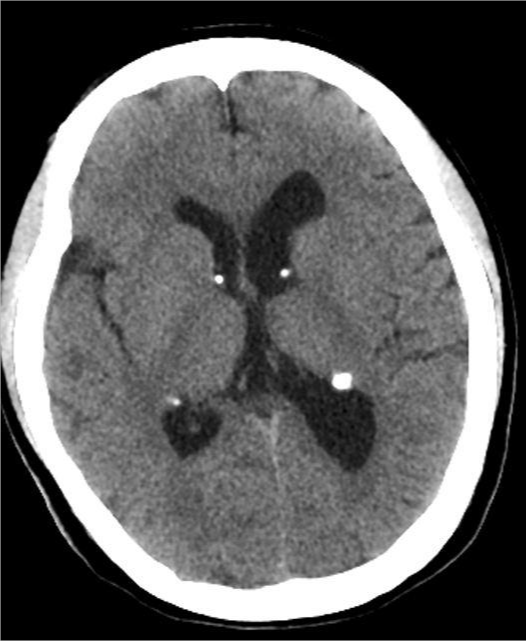

Supplement: hcad125_Supplementary_Data [file hcad125_supplementary_data.zip › suppl_data/sFigure3.png]
